# Role of Galactosylceramide Metabolism in Satellite Glial Cell Dysfunction and Neuron–Glia Interactions in Painful Diabetic Peripheral Neuropathy

**DOI:** 10.3390/cells14060393

**Published:** 2025-03-07

**Authors:** Xin Xu, Yue Zhang, Shuo Li, Chenlong Liao, Xiaosheng Yang, Wenchuan Zhang

**Affiliations:** Department of Neurosurgery, Shanghai Ninth People’s Hospital, School of Medicine, Shanghai Jiao Tong University, Shanghai 200011, China; xuxin1418@163.com (X.X.); yuezhang922@sjtu.edu.cn (Y.Z.); 18451793811@163.com (S.L.); liaochenlong@shsmu.edu.cn (C.L.)

**Keywords:** diabetic peripheral neuropathy (DPN), satellite glial cells (SGCs), galactosylceramide (GalCer), SGC–neuron interactions

## Abstract

Diabetic peripheral neuropathy (DPN) is a prevalent and disabling complication of diabetes, with painful diabetic peripheral neuropathy (PDPN) being its most severe subtype due to chronic pain and resistance to treatment. Satellite glial cells (SGCs), critical for maintaining dorsal root ganglion (DRG) homeostasis, undergo significant structural and functional changes under pathological conditions. This study investigated the role of galactosylceramide (GalCer), a key sphingolipid, in SGC dysfunction and neuron–glia interactions during DPN progression. Using a rat model of PDPN, we employed single-cell RNA sequencing (scRNA-seq), targeted mass spectrometry, and immunofluorescence analysis. The PDPN group exhibited transcriptional activation and structural reorganization of SGCs, characterized by increased SGC abundance and glial activation, evidenced by elevated Gfap expression. Functional enrichment analyses revealed disruptions in sphingolipid metabolism, including marked reductions in GalCer levels. Subclustering identified vulnerable SGC subsets, such as Cluster a, with dysregulated lipid metabolism. The depletion of GalCer impaired SGC-neuron communication, destabilizing DRG homeostasis and amplifying neurodegeneration and neuropathic pain. These findings demonstrate that GalCer depletion is a central mediator of SGC dysfunction in PDPN, disrupting neuron–glia interactions and exacerbating neuropathic pain. This study provides novel insights into the molecular mechanisms of DPN progression and identifies GalCer metabolism as a potential therapeutic target.

## 1. Introduction

Diabetic peripheral neuropathy (DPN) is one of the most common and disabling complications of diabetes, affecting up to 50% of diabetic patients [1]. DPN is characterized by sensory disturbances, pain, and motor deficits, which severely impair patients’ quality of life and contribute to the risk of ulceration and amputation. Among its subtypes, painful diabetic peripheral neuropathy (PDPN) presents the greatest clinical challenge due to the debilitating nature of chronic pain and its resistance to conventional treatments [2]. Despite its prevalence and severity, the lack of understanding of its underlying mechanisms hinders the development of effective therapies.

The pathophysiology of DPN is multifactorial, with hyperglycemia, oxidative stress, and inflammation recognized as major contributors [3,4]. These factors disrupt the homeostasis of the dorsal root ganglion (DRG), a critical structure housing sensory neurons and their closely associated satellite glial cells (SGCs) [5]. SGCs, specialized glial cells that closely envelop neuronal cell bodies in the DRG, play critical roles in maintaining neuronal function by regulating ion homeostasis, buffering neurotransmitters, and providing metabolic and immune support [6,7]. Emerging evidence indicates that SGCs undergo dynamic structural and functional changes under pathological conditions, contributing to neuroinflammation and neuronal dysfunction [8,9]. SGCs share functional similarities with Schwann cells and astrocytes, particularly in their response to injury, neurotransmitter modulation, and calcium signaling. However, SGCs remain distinct in their anatomical localization, molecular profile, and interactions with sensory neurons [5,10]. Despite increasing recognition of SGC involvement in neuropathic pain, their precise role in DPN progression—especially in the context of lipid metabolism and neuron–glia interactions—remains poorly understood.

Among the key processes disrupted in SGCs under pathological conditions, lipid metabolism, particularly sphingolipid metabolism, is increasingly recognized as a critical factor in neuronal and glial function. Galactosylceramide (GalCer), a key sphingolipid component of myelin and neuronal membranes, is essential for maintaining membrane integrity, facilitating signal transduction, and supporting myelin formation [11,12,13]. Aberrant GalCer metabolism compromises membrane integrity, disrupts neuron–glia metabolic exchange, and exacerbates neurodegeneration in DPN [14]. UDP-galactose ceramide galactosyltransferase (UGT8), the rate-limiting enzyme in GalCer biosynthesis, catalyzes the transfer of galactose to ceramide, playing a crucial role in maintaining sphingolipid homeostasis in glial cells [15]. Dysregulation of UGT8 has been implicated in various neuropathological conditions, highlighting its potential role in the disruption of neuron–glia interactions under diabetic neuropathy [16,17]. While lipidomic studies have revealed significant alterations in sphingolipid levels in diabetes, the specific role of GalCer and its biosynthetic enzyme, UGT8, in SGC–neuron interactions and DRG homeostasis remains poorly understood.

To address this gap, we investigated the role of SGCs and GalCer metabolism in the progression of DPN. Using a well-characterized rat model of PDPN, we combined single-cell RNA sequencing (scRNA-seq), targeted mass spectrometry, and immunofluorescence analysis to comprehensively examine the transcriptional heterogeneity of SGC, quantify GalCer levels, and evaluate structural alterations in SGC–neuron interactions. Our study reveals significant metabolic reprogramming in SGCs and highlights the critical role of GalCer in maintaining DRG integrity under neuropathic conditions. These findings provide novel insights into the molecular mechanisms underlying DPN and identify potential therapeutic targets to mitigate disease progression.

## 2. Materials and Methods

### 2.1. Animals

All animal procedures were approved by the Animal Care and Use Committee of Shanghai Ninth People’s Hospital, Shanghai Jiao Tong University School of Medicine, and were conducted in accordance with the guidelines outlined by the National Health and Family Planning Commission of China. Male Sprague–Dawley rats (200–220 g) were obtained from the Shanghai Laboratory Animal Research Center and housed under specific pathogen-free (SPF) conditions in individually ventilated cages. The animals were maintained on a 12 h light–dark cycle with unrestricted access to standard laboratory chow and drinking water.

Prior to any experimental manipulation, the animals were acclimated to the housing conditions for one week. Rats were monitored daily for general health, and their body weights were recorded weekly. All efforts were made to minimize animal discomfort and distress throughout the study. At the end of the experimental period, humane euthanasia was performed using carbon dioxide asphyxiation in accordance with approved guidelines.

### 2.2. Diabetes Induction

Diabetes was induced in male Sprague–Dawley rats by a single intraperitoneal injection of streptozotocin (STZ, 60 mg/kg; Solarbio, Beijing, China) dissolved in freshly prepared 1% citrate buffer (pH 4.5). Control animals received an equivalent volume of the citrate buffer without STZ. To ensure the accuracy of diabetes induction, blood glucose levels were measured from tail vein samples three days post-injection using a glucometer (Bayer HealthCare, Leverkusen, Germany). Rats with fasting blood glucose levels exceeding 16.7 mmol/L were considered diabetic and included in the subsequent experiments.

Throughout the study, diabetic animals were monitored daily for general health and weighed weekly to assess overall physiological status. Control rats were handled and monitored in the same manner to eliminate handling-related variability.

### 2.3. Mechanical Allodynia and Thermal Sensitivity Assessment

The mechanical allodynia assessment has been described in our previous study [18]. Mechanical sensitivity was assessed weekly using von Frey filaments (North Coast, CA, USA) following a standardized up-down method. Rats were placed individually in transparent Plexiglas chambers positioned on an elevated metal mesh floor to allow access to the plantar surface of their hind paws. After a 15 min acclimation period, von Frey filaments of varying forces (1.4, 2, 4, 6, 8, 10, 15, and 26 g-force) were applied perpendicularly to the central region of the hind paw for 5 s, with a 15 s interval between stimulations. The 50% paw withdrawal threshold (PWT) was determined based on Chaplan’s up-down method, which calculates the force at which the animal withdraws its paw in response to stimulation [19]. The hind paw was stimulated only when the rat was stationary and not grooming or exploring. Diabetic rats were classified as exhibiting mechanical allodynia (MA) if their PWT was ≤8 g. Rats with a PWT ≥ 15 g were considered non-MA, while those with intermediate thresholds were excluded from further experiments to ensure consistent group stratification.

Thermal sensitivity was measured using a Hargreaves radiant heat apparatus (IITC Life Science, Woodland Hills, CA, USA) following established protocols [20]. Rats were placed individually in transparent Plexiglas chambers on an elevated glass surface and allowed to acclimate for 15 min. A focused beam of radiant heat was applied to the plantar surface of each hind paw, and withdrawal latency (WL) was recorded as the time taken for the rat to withdraw its paw. The maximum cutoff time was set at 20 s to prevent tissue damage. Each paw was tested three times with an interval of at least 5 min between trials, and the mean latency was used for analysis. We further quantified Heat Hyperalgesia Latency (HHL), calculated as the difference between the control group WL and the WL of the DM or PDPN groups. A higher HHL value indicates increased sensitivity to heat stimuli, reflecting greater hyperalgesia in the Sprague–-Dawley rats.

Following PWT and thermal sensitivity assessments, rats were categorized into three experimental groups: the PDPN group, consisting of diabetic rats with MA (PWT ≤ 8 g), and WL ≤ 10 s; the DM group, composed of diabetic rats without MA (PWT ≥ 15 g), and WL > 10 s; and the Control group, comprising non-diabetic rats without any treatment. These groups were used for all subsequent analyses.

### 2.4. Tissue Dissociation and Single-Cell Preparation

On day 28 post-STZ injection, rats from each experimental group (Control, DM, PDPN) were euthanized, and the bilateral lumbar L5 DRGs were harvested under sterile conditions. DRGs were rinsed three times with Hanks’ Balanced Salt Solution (HBSS; Sigma-Aldrich, Saint Louis, MO, USA) to remove blood and debris. Tissue dissociation was performed using the GEXSCOPETM Tissue Dissociation Solution (Singleron, Nanjing, China) at 37 °C with gentle agitation for 15 min. The resulting suspension was passed through a 40 μm cell strainer to obtain a single-cell suspension. To minimize contamination from red blood cells, the suspension was treated with GEXSCOPETM red blood cell lysis buffer (Singleron, Nanjing, China) at 25 °C for 10 min. Cells were subsequently centrifuged at 500× *g* for 5 min, washed with phosphate-buffered saline (PBS; HyClone, Logan, UT, USA), and resuspended in PBS. Viability and cell counts were determined using trypan blue staining, ensuring > 85% cell viability prior to further analysis.

### 2.5. Library Construction and scRNA-Seq Data Pre-Processing

Single-cell RNA sequencing libraries were constructed using the GEXSCOPETM Single-Cell RNA Library Kit (Singleron, Nanjing, China) following the manufacturer’s protocol. Briefly, single-cell suspensions were loaded onto microfluidic devices along with barcoded beads, ensuring that each microwell captured a single cell. After cell lysis, mRNA was captured and reverse transcribed into cDNA, which was subsequently amplified and processed into sequencing libraries. The libraries were sequenced on an Illumina HiSeq X10 platform with 150 bp paired-end reads to ensure high coverage and depth.

The raw sequencing reads were processed using an in-house pipeline to generate gene expression profiles. Reads were initially subjected to quality control using FastQC, followed by adapter trimming with fastp. The filtered reads were aligned to the RGSC rn6 reference genome using the STAR aligner (v2.5.3a) with ensemble v92 gene annotations. Unique molecular identifier (UMI) counts per gene per cell were calculated with featureCounts (v1.6.2) for transcript quantification. Data quality metrics, including sequencing depth, UMI counts, and gene counts, were evaluated to filter low-quality cells.

Further analysis was conducted using the Seurat package (v5.1.0) in R (v4.4.0). Cells were filtered based on the number of detected genes, total RNA counts, and the percentage of mitochondrial gene expression. Specifically, cells with fewer than 200 or more than 2500 detected genes or with total RNA counts below 1000 were excluded to ensure high-quality data. After filtering, the remaining cells were categorized into experimental groups as follows: 1883 cells from the Control group, 1286 cells from the DM group, and 2532 cells from the PDPN group.

Gene expression data were normalized, log-transformed, and scaled to mitigate technical noise. The top 2000 highly variable genes were identified for downstream analyses. Dimensionality reduction was performed using principal component analysis (PCA), and batch effects were corrected using canonical correlation analysis (CCA). The top 20 principal components were used for clustering, and t-distributed stochastic neighbor embedding (t-SNE) was applied to visualize the clustering results.

The sequencing data for the analyzed cell populations has been deposited in the National Center for Biotechnology Information (NCBI) Gene Expression Omnibus (GEO) database under accession number GSE176017.

### 2.6. Clustering and Cell Type Identification

Clustering and cell type identification were conducted using the Seurat package (v5.1.0) in R. Following data normalization and dimensionality reduction, the top 20 principal components were selected for clustering based on the shared nearest neighbor (SNN) graph. Clusters were identified using the Louvain algorithm, and the resolution parameter was optimized to balance granularity and interpretability. The clustering results were visualized using t-SNE and uniform manifold approximation and projection (UMAP) plots.

Cell type annotation was performed by cross-referencing differentially expressed genes in each cluster with established cell markers. The cell types identified, including SGC, neurons, Schwann cells, vascular endothelial cells (VECs), macrophage, and proliferating satellite glial cells (PSGC), have been reported in our previous study [18]. For detailed cell marker information, please refer to Table S1.

### 2.7. GO and KEGG Pathway Enrichment Analysis

Differentially expressed genes (DEGs) identified from single-cell RNA sequencing data were subjected to Gene Ontology (GO) and Kyoto Encyclopedia of Genes and Genomes (KEGG) pathway enrichment analysis to explore the biological processes and molecular pathways associated with SGC subsets. GO and KEGG enrichment analyses were performed in R (v4.4.0) using the clusterProfiler package (v4.8.0). Gene annotations for GO biological processes, cellular components, and molecular functions were retrieved from the org.Rn.eg.db database (v3.17.0), and KEGG pathway annotations were obtained from the KEGGREST package (v1.40.0). Enrichment results were visualized using the ggplot2 package, with the top 10 enriched terms ranked by adjusted *p*-values for GO and KEGG pathways. The enrichment significance was evaluated based on the Benjamini-Hochberg corrected *p*-value (q-value < 0.05).

### 2.8. Targeted Mass Spectrometry Analysis

Targeted metabolomics analysis was conducted to quantify GalCer levels in DRG samples. Sample preparation involved the homogenization of tissue followed by lipid extraction using a chloroform/methanol (2:1, *v*/*v*) solvent system. Extracted lipids were dried under nitrogen and reconstituted in a solvent mixture compatible with mass spectrometry. The prepared samples were analyzed using liquid chromatography-tandem mass spectrometry (LC-MS/MS) to ensure high specificity and sensitivity for GalCer detection.

The LC-MS/MS analyses were performed by the Beijing ZKGX Research Institute of Science and Technology (Chemical Lab, Beijing, China), following their established protocols. Calibration curves were generated using authentic standards, and internal standards were included to correct for matrix effects and variability. Data acquisition and quantification were conducted using software provided by the instrument manufacturer. The results are reported as relative GalCer levels normalized to tissue weight.

### 2.9. Immunofluorescence Staining

Immunofluorescence staining was performed on frozen DRG sections to detect UGT8 and GFAP expression. Frozen sections were prepared using a cryostat (CryoStar NX50, Thermo Fisher, Waltham, MA, USA) and fixed with a neutral tissue fixation buffer for 30 min at room temperature. After fixation, sections were washed three times with PBS (pH 7.4) on a decolorizing shaker, each wash lasting 5 min. Antigen retrieval was conducted using citrate buffer (pH 6.0) in a water bath at 95 °C for 30 min, followed by natural cooling to room temperature. Sections were washed again with PBS and blocked with 3% bovine serum albumin (BSA) for 30 min at room temperature. Primary antibodies were applied to the sections: rabbit anti-UGT8 (1:100, Abcepta, Soochow, China, AP11630C) and mouse anti-GFAP (1:300, Dako, Copenhagen, Denmark, Z0334). The slides were incubated overnight at 4 °C in a humidified chamber.

Following incubation, sections were washed three times with PBS and incubated with secondary antibodies for 50 min at room temperature in the dark. Alexa Fluor 488-conjugated goat anti-rabbit IgG (green, 1:400, Servicebio, Wuhan, China, GB25303) and CY3-conjugated goat anti-mouse IgG (red, 1:300, Servicebio, Wuhan, China, GB21301) were used as secondary antibodies. Nuclei were counterstained with DAPI (Servicebio, Wuhan, China, G1012) for 10 min at room temperature, followed by quenching of tissue autofluorescence using autofluorescence quenching solution (Servicebio, Wuhan, China, G1221). Slides were mounted with an antifade mounting medium (Servicebio, Wuhan, China, G1401), and images were captured using a Nikon Eclipse C1 upright fluorescence microscope (Nikon, Tokyo, Japan). *UGT8* expression was visualized as green fluorescence, GFAP as red fluorescence, and nuclei as blue fluorescence.

### 2.10. Western Blot

Western blot analysis was performed to assess Ugt8 protein expression in DRG tissue samples from the Control, DM, and PDPN groups. Total protein was extracted using RIPA lysis buffer (Beyotime, Shanghai, China) supplemented with a protease and phosphatase inhibitor cocktail (Beyotime, Shanghai, China). Protein concentration was determined using the BCA Protein Assay Kit (Beyotime, Shanghai, China). Equal amounts of protein (30 µg per sample) were separated via SDS-PAGE (10% gel) and transferred onto PVDF membranes (Millipore, Burlington, MA, USA). After blocking with 5% non-fat milk in TBST (Tris-buffered saline with 0.1% Tween-20) for 1 h at room temperature, membranes were incubated overnight at 4 °C with primary antibodies anti-Ugt8 (1:1000, Abcepta, Soochow, China, AP11630C), anti-Gfap (1:500, Sigma-Aldrich, MO, USA, SAB5700611) and anti-GAPDH (1:5000, Proteintech, Wuhan, China) as a loading control.

The next day, membranes were washed with TBST and incubated with HRP-conjugated secondary antibodies (1:5000, Proteintech, Wuhan, China) for 1 h at room temperature. Bands were visualized using an ECL chemiluminescence detection kit (Thermo Fisher Scientific, MA, USA), and densitometry analysis was performed using ImageJ software (v1.8.0, NIH, Bethesda, MD, USA). Protein expression levels were normalized to GAPDH.

### 2.11. Quantitative Polymerase Chain Reaction (qPCR)

Q-PCR was performed to assess the expression levels of *Ugt8* and *Gfap* in DRG tissues from the Control, DM, and PDPN groups. Total RNA was extracted using the TRIzol reagent (Invitrogen, Carlsbad, CA, USA) according to the manufacturer’s instructions. RNA purity and concentration were determined using a NanoDrop 2000 spectrophotometer (Thermo Fisher Scientific, USA), and samples with an A260/A280 ratio between 1.8 and 2.0 were used for further analysis.

Reverse transcription was performed using the PrimeScript RT reagent kit (Takara, Kobe, Japan) to synthesize complementary DNA (cDNA) from 1 µg of total RNA. qPCR was conducted using TB Green Premix Ex Taq II (Takara, Kobe, Japan) on a QuantStudio 6 Flex Real-Time PCR System (Applied Biosystems, Foster City, CA, USA).

The following gene-specific primers were used:

*Ugt8*
Forward Primer: 5′-AAGACACCAAGACAAAGCCA-3′
Reverse Primer: 5′-GAATTCCCAAGACCCACTCTG-3′
*Gfap*
Forward Primer: 5′-CACCACGATGTTCCTCTTGA-3′
Reverse Primer: 5′-ATCGAGATCGCCACCTACAG-3′
*β-actin*
Forward Primer: 5′-CATGTTTGAGACCTTCAACAC-3′
Reverse Primer: 5′-CCAGGAAGGAAGGCTGGAA-3′

qPCR reactions were amplified in 20 µL reaction volume. Relative gene expression levels were calculated using the 2^−ΔΔ^Ct method, with *β-actin* as the internal control.

### 2.12. Statistical Analysis

All statistical analyses were performed using GraphPad Prism (v8.0.2) and R software (v4.4.0). Data are presented as mean ± SEM unless otherwise specified. For comparisons between multiple groups, statistical significance was determined using one-way or repeated measures two-way analysis of variance (ANOVA), followed by Tukey’s or Bonferroni post hoc tests, as appropriate. For pairwise comparisons, unpaired Student’s *t*-tests were used. Non-parametric tests, such as the Mann–Whitney U test, were employed for data not meeting normality or homogeneity of variance assumptions. Normality was assessed using the Shapiro–Wilk test. For enrichment analyses, the Benjamini–Hochberg method was applied to adjust *p*-values for multiple testing, with an adjusted *p*-value (q-value) < 0.05 considered statistically significant. Visualizations, including box plots, bar graphs, heatmaps, and network diagrams, were generated using ggplot2 (v3.4.0) in R. A *p* value less than 0.05 means there is a statistically significant difference.

## 3. Results

### 3.1. Establishment of the Diabetic Neuropathy Model and Group Stratification

**Figure 1:**
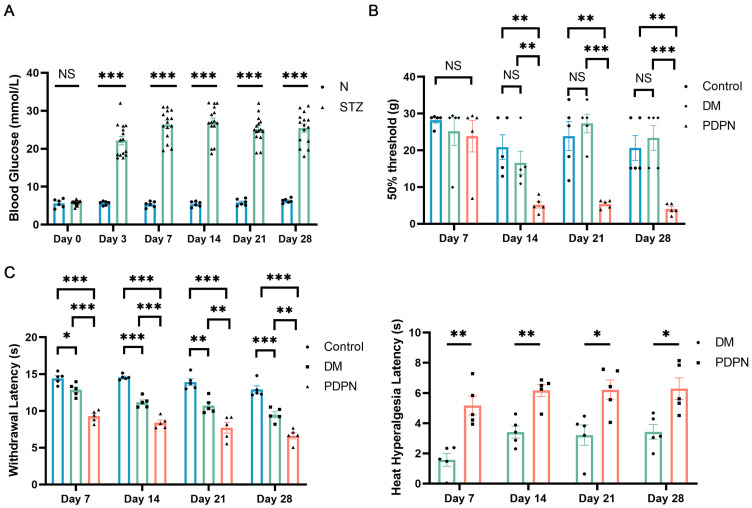
Establishment of the diabetic neuropathy model and group stratification. (**A**) Blood glucose levels in control (N, n = 6) and STZ-treated (n = 15) rats over 28 days. STZ-treated rats developed significant hyperglycemia from Day 3 onward compared to the control group. (**B**) Mechanical sensitivity, measured as the 50% paw withdrawal threshold (PWT), was assessed in control (n = 5) and STZ-treated diabetic rats, which were further stratified into diabetic non-allodynic (DM, n = 5) and diabetic allodynic (PDPN, n = 5) groups based on their pain responses. Significant differences in PWT were observed in the PDPN group compared to the DM and control groups starting from Day 14, demonstrating variability in pain sensitivity among STZ-treated rats. (**C**) Thermal sensitivity assessment using the Hargreaves test. The PDPN group showed significantly reduced withdrawal latency (WL) in response to radiant heat compared to the DM and control groups, indicating thermal hyperalgesia. Heat hyperalgesia latency (HHL) was significantly increased in the PDPN group, further confirming heightened thermal sensitivity. Data are presented as mean ± SEM. * *p* < 0.05, ** *p* < 0.01, *** *p* < 0.001, NS: not significant.

To establish a reliable diabetic neuropathy model, 15 male Sprague–Dawley rats were injected with STZ (60 mg/kg) to induce diabetes, while control animals received citrate buffer. Blood glucose levels were monitored over 28 days post-injection. As shown in Figure 1A, STZ-treated rats developed significant hyperglycemia (≥16.7 mmol/L) as early as day 3, and this condition persisted throughout the experimental period (*p* < 0.001 compared to controls). In contrast, blood glucose levels in the control group remained within the normal range.

Mechanical sensitivity was evaluated weekly using von Frey filaments. By day 14, only five diabetic rats exhibited distinct mechanical allodynia (reduced 50% PWT), which persisted through day 28 (Figure 1B). In the thermal hyperalgesia assessment, these 5 MA diabetic rats also showed a significantly reduced WL in response to radiant heat (WL ≤ 10 s), indicating increased sensitivity to thermal stimuli. HHL values in these rats were markedly elevated, confirming pronounced thermal hyperalgesia.

Based on the PWT and WL, diabetic rats were stratified into two groups: the PDPN group, defined by a PWT ≤ 8 g and WL ≤ 10 s, indicating significant mechanical hypersensitivity; and the DM group, with a PWT ≥ 15 g and WL > 10 s, indicating the absence of mechanical allodynia. Control rats maintained consistently high PWT values, demonstrating the absence of neuropathy.

These findings validate the successful establishment of a diabetic model with pronounced mechanical allodynia and thermal hyperalgesia in STZ-treated rats, creating a reliable foundation for investigating the pathophysiological mechanisms of DPN.

### 3.2. Cellular Composition of DRG Tissue Revealed by Single-Cell RNA Sequencing

**Figure 2:**
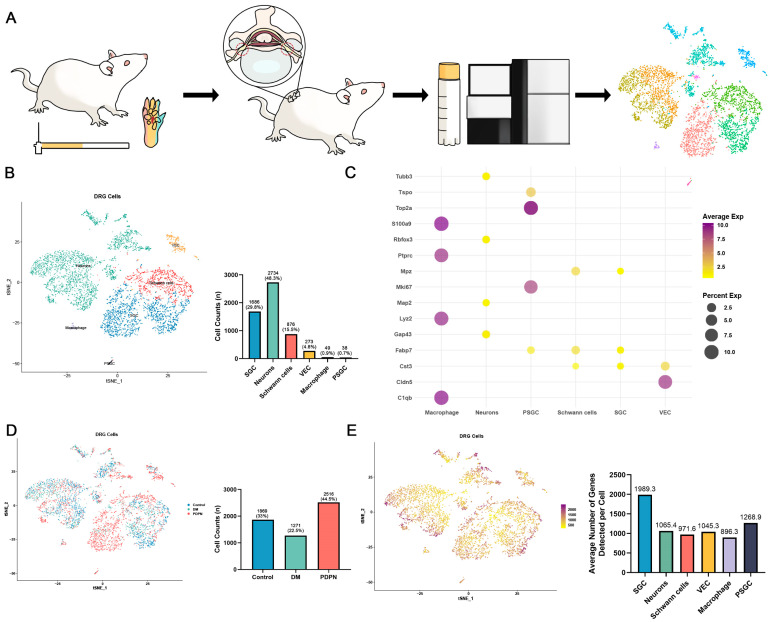
Comprehensive Analysis of Cell Types and Gene Expression in DRG Tissues. (**A**) Schematic illustration of the experimental workflow. The diagram shows the process of collecting dorsal root ganglion (DRG) tissues from rats, followed by cell dissociation, single-cell RNA sequencing, and subsequent data analysis including clustering and visualization of different cell populations. (**B**) t-SNE plot and cell count bar graph of different cell types identified in DRG tissues. The t-SNE plot shows the clustering of different cell types, including SGCs (Satellite Glial Cells), neurons, Schwann cells, vascular endothelial cells (VECs), macrophage, and proliferating satellite glial cells (PSGC). The bar graph displays the number of cells in each cluster, with SGCs (1688, 29.8%), neurons (2734, 48.3%), Schwann cells (876, 15.5%), VECs (273, 4.8%), macrophage (49, 0.9%), and PSGC (38, 0.7%). (**C**) Dot plot showing the expression of selected marker genes across different cell types. The size of the dots represents the percentage of cells expressing the gene, and the color intensity indicates the average expression level. (**D**) t-SNE plot and cell count bar graph showing the distribution of all DRG cells across three groups (Control, DM, PDPN). The t-SNE plot visualizes the clustering of DRG cells from different conditions. The bar graph displays the total number of DRG cells identified in each group, with Control (1869 cells, 33%), DM (1271 cells, 22.5%), and PDPN groups (2516 cells, 44.5%). (**E**) t-SNE plot and bar graph illustrating the number of detected genes per cell across different DRG cell populations. The t-SNE plot visualizes the distribution of DRG cells based on the number of genes detected per cell, with darker shades representing a higher number of detected genes. The bar graph on the right quantifies the average number of detected genes per cell within each DRG cell type. SGCs exhibited the highest average number of detected genes (1989.3 per cell), followed by neurons (1065.4), Schwann cells (971.6), VECs (1045.3), macrophages (896.3), and PSGC (1268.9).

To investigate the cellular landscape of DRG in diabetic neuropathy, scRNA-seq was performed on DRG tissues from the Control, DM, and PDPN groups. The experimental workflow is depicted in Figure 2A, including tissue extraction, single-cell preparation, sequencing, and bioinformatic analyses.

Single-cell clustering identified six major cell types in the DRG (Figure 2B), including SGCs (1688 cells, 29.8%), neurons (2734 cells, 48.3%), Schwann cells (876 cells, 15.5%), VECs (273 cells, 4.8%), macrophage (49 cells, 0.9%), and PSGC (38 cells, 0.7%). The distribution of these cell types highlights the complexity of the DRG microenvironment. Cluster-specific markers were used to validate cell type identities (Figure 2C; marker information detailed in Table S1), confirming the reliability of our clustering approach.

Further analysis of the DRG cellular composition revealed differences in the proportion of detected cells among the three groups. The PDPN group had the highest proportion of DRG cells (2516 cells, 44.5%), followed by the Control group (1869 cells, 33%) and the DM group (1271 cells, 22.5%) (Figure 2D). These variations suggest potential changes in cellular homeostasis within the DRG during PDPN progression. Then, we analyzed the number of genes detected per cell within each DRG subpopulation. Grouping cells by DRG subclusters revealed that SGCs exhibited the highest average number of detected genes among all cell types (Figure 2E). This observation suggests that SGCs, as key glial cells in the DRG, may participate in a broader range of biological functions compared to other cell types. Gene expression analysis revealed differential expression patterns for key markers across cell types (Figure S1). *Gap43* and *Fabp7*, associated with neural and glial functions, showed elevated expression in SGCs, whereas *Mpz* and *Cldn5* were enriched in Schwann cells and vascular endothelial cells, respectively. In addition, *Top2a* was highly enriched in PSGC, while *Lyz2* was prominently expressed in macrophages, reflecting their proliferative and immune-related functions, respectively.

These results illustrate the complex cellular landscape of the DRG and highlight significant alterations in cellular composition during PDPN progression. The abundant gene expression patterns observed in SGCs suggest their pivotal role in maintaining DRG homeostasis and contributing to the pathophysiology of neuropathy.

### 3.3. Altered SGC Composition and Functional Enrichment in Diabetic Neuropathy

**Figure 3:**
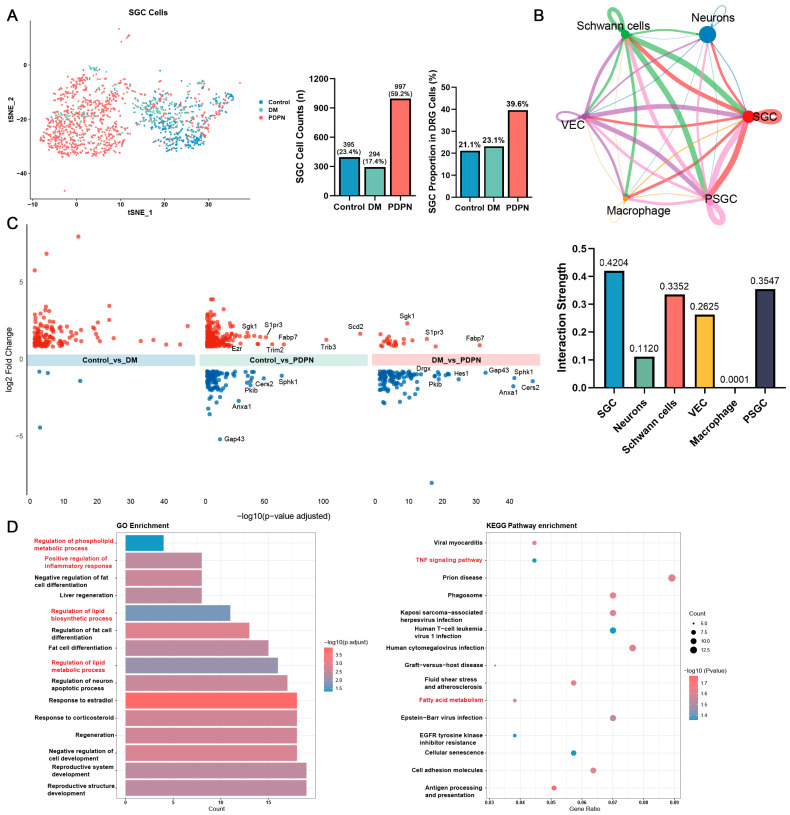
Analysis of SGC cells and Their Associated Pathways. (**A**) t-SNE plot of SGC cells color-coded by three groups (Control, DM, PDPN). The middle bar graph displays the total SGC cell counts for each group, with 395 cells (23.4%) in the Control, 294 cells (17.4%) in the DM, and 997 cells (59.2%) in the PDPN group. The right bar graph shows the proportion of SGC cells relative to the total DRG cell population in each group, with SGC accounting for 21.1% of DRG cells in the Control, 23.1% in the DM, and 39.6% in the PDPN group. (**B**) CellChat interaction network illustrating the weight of interactions among different cell types. The network highlights interactions between SGCs cells and other cell types such as Schwann cells, neurons, vascular endothelial cells (VECs), macrophage, and proliferating satellite glial cells (PSGC); Edge thickness represents the interaction frequency, with SGCs exhibiting strong interactions with neurons (top panel). The bar graph on the bottom quantifies the interaction strength between each cell type and neurons, showing that SGCs have the highest communication strength (0.4204), followed by PSGC (0.3547), neurons (0.1120), Schwann cells (0.3352), VECs (0.2625), and macrophages (0.0001). (**C**) Volcano plot of differentially expressed genes (DEGs) in SGCs across the three experimental groups. Upregulated genes in the Control vs. DM, Control vs. PDPN, and DM vs. PDPN comparisons are shown in red, while downregulated genes are in blue. (**D**) Enrichment analysis of differentially expressed genes. The left panel shows the GO enrichment results, and the right panel shows KEGG pathway enrichment results. The bar colors in the GO enrichment chart represent the −log10 adjusted *p*-values, and the sizes of the dots in the KEGG enrichment chart indicate the gene counts within each pathway, with colors representing the −log10 adjusted *p*-values.

To further investigate the role of SGCs in DPN, we analyzed SGC-specific clustering and functional changes across the Control, DM, and PDPN groups. The t-SNE plot of SGCs revealed distinct clustering patterns among the groups, with the PDPN group exhibiting the highest proportion of SGC cells (997 cells, 59.2%) compared to the Control (395 cells, 23.4%) and DM (294 cells, 17.4%) groups. When normalized to the total DRG cell population, SGCs accounted for 39.6% of DRG cells in the PDPN group, compared to 21.1% in the Control and 23.1% in the DM group (Figure 3A). The progressive expansion of SGCs further suggests their potential involvement in PDPN progression.

Cell–cell interaction networks, constructed using CellChat (v1.6.1), showed that SGCs had stronger communication with neurons compared to other cell types across all groups (Figure 3B). Differential gene expression analysis was conducted using the criteria of *p* value < 0.05 and absolute value of average log_2_(Foldchange) > 1, which identified 103 significant DEGs in Control vs. DM, 257 in Control vs. PDPN, and 90 in DM vs. PDPN comparisons. During the progression to PDPN, SGCs exhibited significant upregulation of genes associated with inflammation, lipid metabolism, and cellular stress responses (Figure 3C). Notable genes such as *S1pr3*, *Fabp7*, and inflammatory response regulators were enriched, suggesting metabolic alterations in SGCs under neuropathic conditions. However, lipid metabolism-related genes *Cers2* and *Sphk1* were significantly downregulated, indicating impaired sphingolipid metabolism in PDPN.

GO enrichment analysis highlighted biological processes such as regulation of lipid and phospholipid biosynthetic and metabolic processes, as well as positive regulation of inflammatory response as significantly enriched in the PDPN group (Figure 3D, left panel). KEGG pathway analysis identified fatty acid degradation, and TNF signaling pathways as the most prominently altered (Figure 3D, right panel).

The above results suggest that SGCs in the PDPN group undergo metabolic and inflammatory biological processes and participate in the pathogenesis of DPN through crosstalk with neurons.

### 3.4. Subclustering of SGC Reveals Functional Diversity and Dynamic Changes in Diabetic Neuropathy

**Figure 4:**
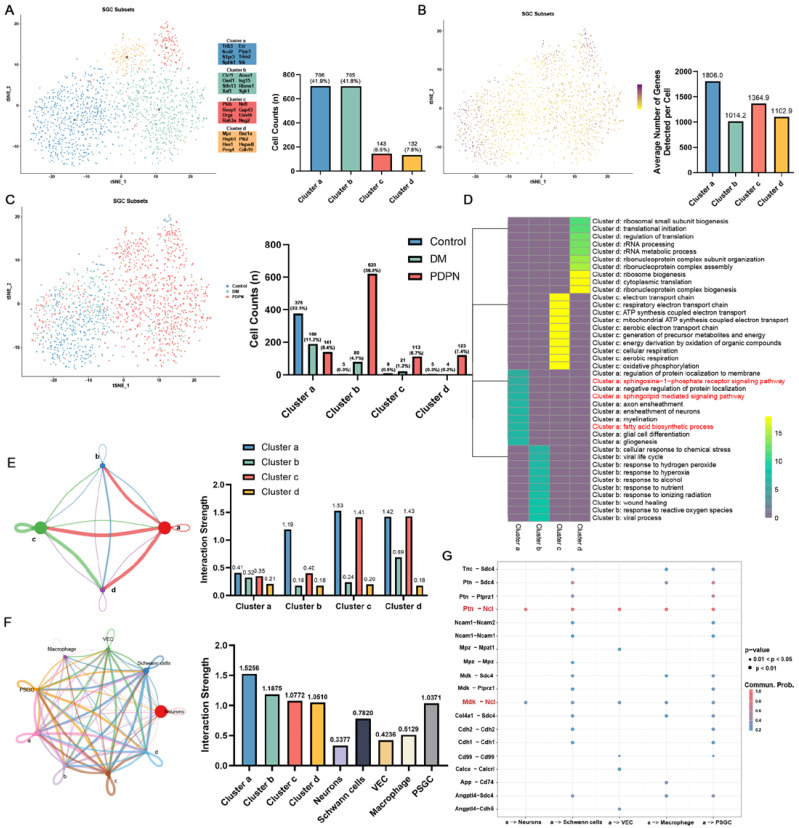
Subcluster Analysis of SGC Cells and Interaction Networks. (**A**) t-SNE plot showing the clustering of SGCs (Satellite Glial Cells) subclusters labeled as a, b, c, and d. The bar graph on the right shows the cell counts for each subcluster: Cluster a (706 cells, 41.9%), Cluster b (705 cells, 41.8%), Cluster c (143 cells, 8.5%), and Cluster d (132 cells, 7.8%). (**B**) t-SNE plot colored by the number of genes expressed in each SGC cell, highlighting the distribution of cells within the t-SNE space. The color intensity represents the number of genes expressed in each cell. The bar graph on the right quantifies the average number of detected genes per cell across different cell populations, showing that Cluster a exhibits the highest transcriptional activity (1806 genes per cell), followed by Cluster b (1014.2), Cluster c (1364.9) and Cluster d (1102.9). (**C**) t-SNE plot of SGC subclusters across experimental groups (Control, DM, PDPN). The bar graph on the right displays the number of SGC cells in each subcluster for each group: Control, DM, and PDPN. (**D**) Heatmap showing GO enrichment analysis for each SGC subcluster. The heatmap displays the significant GO terms associated with each subcluster, highlighting pathways related to sphingolipid metabolism and fatty acid biosynthesis. (**E**) CellChat interaction network within SGC subclusters. The network diagram illustrates the interactions among subclusters a, b, c, and d; Edge thickness represents the interaction frequency, with SGCs exhibiting strong interactions with neurons (left panel). The bar graph on the right quantifies the strength of the interactions between each cell type, showing that Cluster a has the highest relative communication strength with other cell clusters, Cluster b (1.19), Cluster c (1.53), and Cluster d (1.42). (**F**) CellChat interaction network between SGC subclusters and other cell types, including neurons, Schwann cells, vascular endothelial cells (VECs), macrophage, and proliferating satellite glial cells (PSGC). The network diagram shows the interactions, with line thickness representing the interaction strength. The bar graph on the right quantifies the interaction strength between each cell type and neurons, showing that Cluster a has the highest relative communication strength with neurons (1.5256). (**G**) Dot plot displaying the expression of specific ligand-receptor pairs across different cell types. The size of the dots represents the percentage of cells expressing the ligand-receptor pair, and the color intensity indicates the average expression level, highlighting the ligand-receptor pairs of Cluster a and neurons.

To further explore the heterogeneity within the SGC population, we extracted SGC cells from DRG scRNA-seq data and performed subclustering analysis, identifying four distinct SGC subsets (Figure 4A). Among these, Cluster a emerged as the largest subset (705 cells, 41.9%), exhibiting unique transcriptional features associated with lipid metabolism and neuronal support. Genes such as *S1pr3* and *Sphk1*, involved in sphingolipid metabolism, suggest a regulatory role of Cluster a in lipid signaling pathways, which are critical for cellular homeostasis under neuropathic conditions. Additionally, *Trim2* and *Ezr*, known for their functions in cytoskeletal organization and neural development, highlight Cluster a’s role in maintaining neuronal structure and promoting axonal integrity. The expression of *Scd2* and *Plpp3*, regulators of lipid biosynthesis and phospholipid metabolism, further supports Cluster a’s metabolic specialization.

Analysis of the distribution of SGC subsets across the Control, DM, and PDPN groups revealed significant shifts in their composition (Figure 4C). Cluster a exhibited a progressive reduction in cell numbers as the disease advanced, with the lowest count observed in the PDPN group. This decline suggests a functional loss of Cluster a during diabetic neuropathy, which may impair lipid homeostasis and neuronal support functions, exacerbating disease pathology. Conversely, Clusters b, c, and d displayed compensatory expansion in the PDPN group, contributing to the overall increase in SGC numbers. Cluster b, which exhibits a proliferative transcriptional profile, showed a marked increase, suggesting enhanced cellular replication in response to neuropathic stress. Clusters c and d, enriched for inflammatory and stress-response genes, also expanded, reflecting their potential adaptive mechanisms to counteract the pathological microenvironment in the DRG.

These findings demonstrate a dynamic shift in SGC subset composition during diabetic neuropathy, with the decline of Cluster a and the compensatory expansion of Clusters b, c, and d contributing to a restructured glial environment that likely influences disease progression.

### 3.5. Functional Enrichment and Interaction Analysis of SGC Subsets

To further explore the functional diversity of SGC subsets, we conducted transcriptional activity analysis, GO enrichment, and cell-cell communication analysis. The number of expressed genes per cell varied significantly among subsets, with Cluster a exhibiting the highest transcriptional activity, whereas Clusters b, c, and d showed relatively similar levels of transcriptional activity (Figure 4B). This suggests that Cluster a is the most metabolically active subset, likely playing a central role in lipid metabolism, cellular signaling, and neuronal support functions. In contrast, Clusters b, c, and d may be more specialized in stress adaptation, immune modulation, and glial reactivity under neuropathic conditions. GO enrichment analysis revealed distinct biological functions for each subset (Figure 4D). Cluster a was significantly enriched in sphingolipid metabolism-related processes, including fatty acid biosynthetic process, sphingosine-1-phosphate receptor signaling pathways, and sphingolipid-mediated signaling pathways. This is supported by the high expression of key sphingolipid metabolism regulators such as *Cers2*, *S1pr3*, and *Sphk1*, suggesting that Cluster a is essential for maintaining lipid homeostasis in the DRG. The progressive loss of Cluster a in PDPN may lead to disruptions in lipid metabolic balance, exacerbating glial dysfunction and neuronal stress.

Cell–cell communication analysis further underscored the importance of Cluster a in the SGC network. Cluster a exhibited the strongest interaction strength within the SGC network, reinforcing its role as a key hub in metabolic regulation and cellular signaling (Figure 4E). Furthermore, neuron–SGC interactions were also prominent, with neurons displaying the strongest communication links with Cluster a, indicating its crucial involvement in maintaining neuron–glia interactions and neuronal support mechanisms (Figure 4F). Ligand-receptor analysis revealed that Cluster a established specific communication with neurons through key signaling pairs, including Ptn–Ncl and Mdk–Ncl, which regulate neuronal repair, glial function, and neuroprotection (Figure 4G). These findings highlight Cluster a as a major mediator of neuron-glia crosstalk in DRG homeostasis.

All the above results highlight that Cluster a plays a pivotal role in lipid metabolism, neuron-glia communication, and glial network coordination. Its enrichment in sphingolipid metabolism pathways, strong internal connectivity, and direct interactions with neurons underline its essential role in maintaining DRG homeostasis. The progressive depletion of Cluster a in PDPN indicates a potential loss of its neuroprotective and metabolic regulatory functions, which may contribute to exacerbated neuronal stress, impaired glial homeostasis, and worsening neuropathic pain.

### 3.6. GalCer Levels Are Significantly Reduced in PDPN and Correlate with SGC–Neuron Interactions

**Figure 5:**
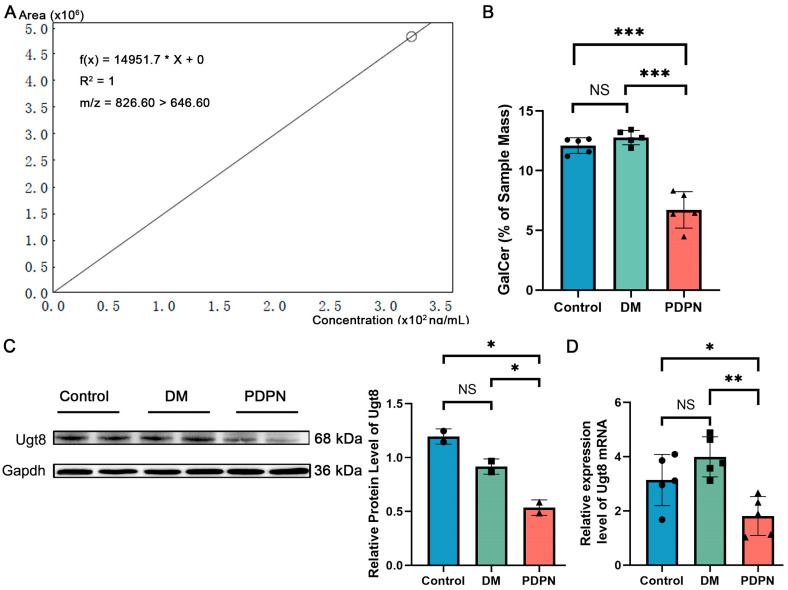
Quantification of GalCer levels in DRG samples using targeted mass spectrometry. (**A**) Standard curve for GalCer quantification, generated using external standards with concentrations ranging from 0 to 3.5 × 10^2^ ng/mL. The curve demonstrates a linear relationship with the equation *f*(*x*) = 14951.7 × *x* + 0 and R^2^ = 1, indicating high precision and reliability. The quantification was based on the ion transition *m*/*z* = 826.60 > 646.60. (**B**) Quantification of GalCer levels (% of sample mass) in the Control, DM, and PDPN groups (n = 5 samples per group). GalCer levels were significantly reduced in the PDPN group compared to the Control and DM groups. (**C**) Western blot analysis of Ugt8 protein expression in DRG samples from Control, DM, and PDPN groups (n = 2 samples per group). The right panel shows the quantification of Ugt8 protein levels normalized to Gapdh. (**D**) qPCR analysis of *Ugt8* mRNA expression in DRG samples from Control, DM, and PDPN groups (n = 5 samples per group). Data are presented as mean ± SEM. * *p* < 0.05, ** *p* < 0.01, *** *p* < 0.001, NS: not significant.

GalCer, a sphingolipid involved in membrane stability and myelin formation, plays a critical role in SGC–neuron interactions, particularly in maintaining neuronal function under stress conditions. To further investigate the metabolic changes in diabetic neuropathy, we quantified GalCer levels in DRG samples across the Control, DM, and PDPN groups using targeted mass spectrometry. A standard curve was generated with external GalCer standards, showing a strong linear relationship (Figure 5A). The calibration equation *f*(*x*) = 14951.7 × *x* + 0 with R^2^ = 1 indicated excellent quantification precision and accuracy within the standard range (0–3.5 × 10^2^ ng/mL). The ion transition *m*/*z* = 826.60 > 646.60 was used to specifically detect and quantify GalCer, ensuring the reliability of the analysis.

Quantitative results revealed significant differences in GalCer levels among the three experimental groups (Figure 5B). While the Control and DM groups exhibited comparable GalCer levels (NS, not significant), the PDPN group showed a marked reduction in GalCer content (*p* < 0.001) compared to both the Control and DM groups. These data are consistent with the dysregulation of lipid metabolism observed in the PDPN group. Detailed GalCer concentrations for each sample are provided in Table S2.

UGT8 is a key enzyme in GalCer biosynthesis, we assessed its protein and mRNA expression levels using Western blot and qPCR [21]. Western blot analysis revealed that Ugt8 protein expression was significantly reduced in the PDPN group compared to the Control and DM groups (*p* < 0.05) (Figure 5C). Similarly, qPCR analysis demonstrated a significant downregulation of *Ugt8* mRNA expression in the PDPN group (*p* < 0.01 vs. DM; *p* < 0.05 vs. Control), further supporting the notion that impaired GalCer biosynthesis is associated with PDPN pathology (Figure 5D).

Given the pivotal role of SGCs in maintaining neuronal homeostasis and lipid metabolism, the observed reduction in GalCer levels is likely associated with SGC dysfunction. Single-cell transcriptomic analysis revealed significant metabolic alterations in SGC from the PDPN group, including downregulation of genes involved in sphingolipid metabolism, such as *Cers2* and *Sphk1* (Figure 3C). Moreover, the enrichment of sphingolipid-related pathways in SGCs subcluster Cluster a, which showed a progressive decline in the PDPN group (Figure 4C,D), further suggests that SGC-mediated GalCer metabolism is impaired under neuropathic conditions.

The above findings suggest that the reduction in GalCer levels in the DRG may stem primarily from SGC dysfunction rather than global alterations in other DRG cell populations. The disrupted GalCer synthesis and metabolism in SGCs likely contribute to impaired neuron–glia interactions, exacerbating DRG instability and neuropathic pain in PDPN.

### 3.7. Structural Alterations and Activation of SGCs in Diabetic Neuropathy

**Figure 6:**
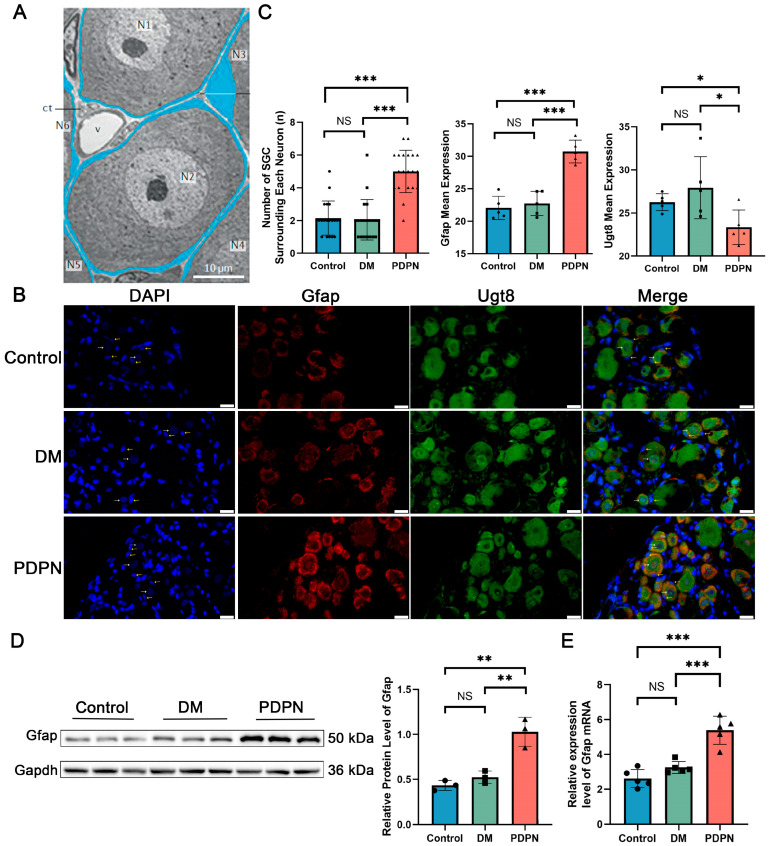
Structural and functional changes in SGC–neuron units and Gfap expression in DRG across Control, DM, and PDPN groups. (**A**) Low-power electron micrograph illustrating the structure of neuron–satellite glial cell (SGC) units in a dorsal root ganglion (DRG). Neurons are labeled as N1–N6, and SGCs are colored in blue. The widened area in the SGCs surrounding N3 contains the cell’s nucleus. ct, connective tissue space; v, blood vessels. Scale bar, 10 µm. Adapted from Hanani M. (2020) [8]. (**B**) Immunofluorescence images of DRG tissue from Control, DM, and PDPN groups, showing DAPI (blue), Gfap (red), and Ugt8 (green). White arrows indicate neuronal nuclei, characterized by their large size and faint appearance, while yellow arrows indicate SGC nuclei, which are smaller and brighter. Scale bars, 10 μm. (**C**) Quantification of the number of SGC nuclei surrounding each neuron. The Control group showed 2.15 ± 1.01 SGCs per neuron, the DM group showed 2.05 ± 1.20, and the PDPN group exhibited a significant increase to 5.00 ± 1.26. The middle panel quantifies Gfap expression, which was significantly elevated in the PDPN group compared to the Control and DM groups. The right panel quantifies Ugt8 expression, showing a significant reduction in the PDPN group. (**D**) Western blot analysis of Gfap protein expression in DRG samples from Control, DM, and PDPN groups (n = 3 samples per group). The right panel shows the quantification of Gfap protein levels normalized to Gapdh. (**E**) qPCR analysis of *Gfap* mRNA expression in DRG samples from Control, DM, and PDPN groups (n = 5 samples per group). Data are presented as mean ± SEM. * *p* < 0.05, ** *p* < 0.01, *** *p* < 0.001, NS: not significant.

SGCs play a critical role in maintaining the microenvironment and functional support of neurons in the DRG. In 2020, Professor Menachem Hanani from the Hebrew University provided detailed insights into the structural relationship between SGCs and neurons in the DRG and the changes in SGCs under the pathological state of DPN. As illustrated in Figure 6A, SGCs closely surround neuronal cell bodies, forming a structural unit. Each neuron is typically enveloped by a thin layer of SGCs, which are connected to each other through specialized gap junctions. The SGC layer acts as a critical regulator, modulating neuronal excitability, maintaining ion homeostasis, and participating in metabolic exchange. SGCs also play an important role in the inflammatory response under the pathological conditions of DPN. In animal models of diabetic neuropathy, SGCs exhibit significant activation features, including upregulated expression of glial fibrillary acidic protein (GFAP), increased gap junction coupling between SGCs and between SGCs and neurons, heightened sensitivity to adenosine triphosphate (ATP), downregulation of potassium ion channel Kir4.1, and increased synthesis and release of pro-inflammatory cytokines. These structural and functional alterations suggest that SGC activation contributes to the pathophysiological processes underlying DPN by enhancing neuron–glia interactions, amplifying neuroinflammation, and modulating neuronal excitability [8].

In the present study, structural and functional alterations in SGC–neuron interactions were analyzed across Control, DM, and PDPN groups using immunofluorescence analysis (Figure 6B). Distinct differences in the arrangement and activation of SGCs were observed among the groups. Neuronal nuclei, labeled with DAPI, appeared large and faintly stained, while SGC nuclei were smaller and exhibited brighter fluorescence. Quantification of SGC nuclei surrounding each neuron revealed a significant increase in the PDPN group compared to the Control and DM groups (*p* < 0.001) (Figure 6C, left panel), and the expression of Gfap, an indicator of SGC activation, was significantly upregulated in the PDPN group (*p* < 0.001) (Figure 6C, middle panel). Western blot and qPCR analyses further confirmed the significant elevation of Gfap expression in the PDPN group compared to the Control and DM groups (Figure 6D,E), indicating enhanced SGC proliferation or activation in response to diabetic neuropathy.

To investigate GalCer synthesis within the DRG, Ugt8, a key enzyme in the GalCer biosynthetic pathway, was used as a marker. Immunofluorescence analysis demonstrated that Ugt8 expression was not restricted to neurons but also colocalized with Gfap (Figure 6B). In neurons, Ugt8 was diffusely distributed within the cytoplasm, whereas in SGC, it was concentrated in Gfap-labeled regions surrounding neuronal cell bodies. This pattern indicates that both neurons and SGCs contribute to GalCer metabolism within the DRG microenvironment. Quantitative analysis of Ugt8 expression revealed a significant reduction in the PDPN group compared to the Control and DM groups (*p* < 0.05) (Figure 6C, right panel), further validating the findings from Western blot and qPCR analyses (Figure 5C,D). The colocalization of Ugt8 with Gfap highlights the metabolic reprogramming of SGCs in response to neuropathic conditions, suggesting that activated SGCs may play a pivotal role in GalCer synthesis and its associated functions.

The above results highlight the critical structural and functional changes in SGCs during diabetic neuropathy, including increased proliferation and activation. The enhanced Gfap expression and elevated SGC–neuron interactions in the PDPN group suggest a compensatory response to neuronal stress and inflammation.

## 4. Discussion

This study provides novel insights into how SGCs dysfunction and GalCer metabolism drive DPN progression. Using an integrative approach combining scRNA-seq, targeted mass spectrometry, and immunofluorescence, we have characterized critical changes in SGC structure and metabolism within the DRG under neuropathic conditions. Our findings reveal that SGC dysfunction, driven by altered lipid metabolism and impaired neuron–glia interactions, is a major driver of PDPN progression.

The dynamic changes observed in SGCs during PDPN underscore their critical role in maintaining DRG homeostasis under pathological conditions. Single-cell transcriptomic profiling, combined with subsequent experimental validation, revealed a marked increase in SGC abundance and transcriptional activation, accompanied by structural reorganization, including an increased number of SGC nuclei surrounding each neuron. In 2020, Hanani et al. have reported that in pathological conditions such as nerve damage, inflammation, and chronic pain, SGCs undergo significant structural and functional changes, transitioning from a resting “flattened” morphology to a more hypertrophic and reactive state. This transformation is characterized by an increase in SGC numbers, enhanced neuronal encapsulation, and upregulated GFAP expression, all of which are hallmarks of glial activation in neuropathic conditions [8]. Our findings further substantiate these structural alterations in the context of PDPN, demonstrating a significant expansion of SGC populations and increased Gfap expression, consistent with prolonged glial activation. Glial activation is a hallmark of neuropathic pain and neuroinflammatory conditions [9]. Our results provide new insights into the specific structural and functional changes in SGCs during PDPN, supporting their maladaptive role in disease progression.

SGCs exhibit functional and molecular similarities with Schwann cells and astrocytes, particularly in their roles in neuroprotection, metabolic support, and inflammatory responses. However, unlike Schwann cells, which myelinate peripheral axons, SGCs primarily ensheath neuronal soma in the DRG and rely on distinct signaling mechanisms [10]. Similarly, while astrocytes contribute to synaptic regulation in the central nervous system, SGCs modulate peripheral neurotransmitter homeostasis [5]. The increased SGC–neuron interactions observed in this study may initially serve as a compensatory response to neuronal damage. Over time, however, prolonged activation disrupts the delicate balance of neuron-glia communication, shifting SGCs from a protective role to a maladaptive state [22,23,24]. This aligns with the concept that glial activation in chronic neuropathic conditions often transitions into sustained inflammation, exacerbating neurodegeneration and disease progression [25].

Functional enrichment analyses reveal that lipid metabolism, particularly sphingolipid pathways, is central to SGC dysfunction in PDPN. GO and KEGG analyses identified significant enrichment of genes such as *S1pr3*, *Sphk1*, and *Cers2*, which regulate lipid signaling and stress responses [26,27,28]. These findings suggest that SGCs attempt to adapt to metabolic stress through lipid reprogramming. However, the observed reduction in GalCer levels indicates the failure of these compensatory mechanisms. Furthermore, STZ-induced upregulation of pyruvate dehydrogenase kinases (PDK2/4) in SGC, neurons, and infiltrating macrophages contributes to sustained glial activation, metabolic stress, and neuroinflammation [8,29]. This cascade further sensitizes sensory neurons, exacerbating neuropathic pain and worsening neuron-glia communication deficits. The depletion of lipid metabolism-enriched SGC subpopulations, such as Cluster a, highlights the vulnerability of specific SGC subsets to metabolic dysregulation, leading to impaired membrane stability, intracellular signaling, and myelin formation. Our findings align with prior studies implicating sphingolipid dysregulation in neurodegenerative and inflammatory conditions, while uniquely emphasizing the critical role of SGCs in mediating these metabolic changes within the context of DPN [30,31].

Among the highly variable genes in Cluster a, *S1pr3* (sphingosine-1-phosphate receptor 3) is particularly notable due to its role in vascular endothelial regulation and angiogenesis. S1P, the ligand for S1PR3, is a bioactive lipid derived from sphingolipid metabolism that influences cell survival, immune modulation, and vascular integrity [32]. The high variability of *S1pr3* in Cluster a suggests that SGCs may leverage S1P/S1PR3-mediated signaling as an adaptive mechanism to regulate metabolic stress and neuroinflammatory responses in PDPN. While S1PR signaling is classically associated with vascular function, there is evidence that S1P signaling also modulates glial activation and neuroinflammation, processes that are central to neuropathic pain pathogenesis [33]. The upregulation of *S1pr3* in SGCs may reflect an attempt to compensate for lipid metabolism disturbances, facilitating glial resilience under metabolic stress. Interestingly, S1PR3-mediated signaling may also intersect with GalCer metabolism, as sphingolipid intermediates such as sphingosine and ceramide are direct precursors for both S1P and GalCer biosynthesis [13]. This suggests that SGCs may upregulate *S1pr3* in response to GalCer depletion, attempting to modulate lipid turnover and maintain homeostasis. However, if this compensatory mechanism fails, the progressive decline in GalCer levels may further disrupt neuron-glia communication, exacerbating DRG instability, chronic neuroinflammation, and neuropathic pain.

GalCer, a pivotal sphingolipid, is crucial for maintaining SGCs and neuronal integrity [11,34]. As a key component of lipid signaling, GalCer supports membrane integrity, facilitates signal transduction, and contributes to the metabolic exchange necessary for DRG homeostasis [12,13,14]. In this study, targeted mass spectrometry showed that GalCer levels were significantly decreased in the PDPN group, and similarly, Western blot and qPCR showed that its synthase Ugt8 was expressed at a reduced level in the PDPN group, highlighting the role of GalCer in driving DPN neuropathy. This reduction likely disrupts SGC function by impairing their ability to buffer extracellular ions, regulate neurotransmitter levels, and provide metabolic support to neurons [16,17,35]. Furthermore, GalCer depletion may destabilize neuron–SGC interactions, leading to compromised glial activation and exacerbating neuroinflammatory responses [36,37]. The loss of GalCer also appears to have a cascading effect on SGC subpopulations, as evidenced by the depletion of lipid metabolism-enriched Cluster a. This suggests that specific SGC subsets may be particularly vulnerable to metabolic dysregulation, amplifying the pathological cascade in PDPN. While previous studies have broadly implicated sphingolipid metabolism in diabetes-related complications, our findings uniquely highlight GalCer as a central mediator of SGC dysfunction in DPN [38]. The destabilization of GalCer-dependent pathways likely impairs SGCs’ capacity to maintain neuronal homeostasis, shifting their role from a protective to a maladaptive state that perpetuates neuropathic pain and DRG dysfunction [25].

Neuron–SGC interactions are fundamental to maintaining DRG homeostasis, particularly under pathological conditions such as PDPN [8]. Ligand-receptor analysis revealed robust signaling activity, with pathways such as Ptn–Ncl and Mdk–Ncl playing critical roles in neuronal repair and signal transduction [39]. The disruption of these pathways in the PDPN group likely increases neuronal vulnerability, impairs axonal maintenance, and perpetuates neuroinflammation. GalCer depletion further destabilizes these interactions by impairing SGC activation and intracellular signaling, initiating a pathological cascade that amplifies neuropathic pain and DRG dysfunction.

Despite the strengths of this study, several limitations warrant discussion. First, while the rat model of PDPN provides valuable insights into the mechanisms of diabetic neuropathy, species-specific differences may limit the direct translation of these findings to humans, necessitating validation in human tissues or advanced organoid models. Second, although single-cell RNA sequencing provides valuable insights into transcriptional regulation, due to factors such as post-transcriptional modifications, translation efficiency, and protein degradation, we should also integrate multi-omics approaches, including proteomics and metabolomics, to comprehensively assess the significance of the functional significance of transcriptome changes. Third, while we demonstrated a strong association between reduced GalCer levels and SGC dysfunction, the causal relationship remains to be elucidated. Further investigation into the upstream regulators of GalCer metabolism and their downstream effects on SGC–neuron interactions will be critical for establishing mechanistic links. Lastly, our study primarily focused on SGCs and their interactions with neurons, leaving the roles of other cell types, such as Schwann cells and macrophage, underexplored. Future studies addressing these gaps will provide a more comprehensive understanding of DRG pathology in DPN.

## 5. Conclusions

This study identifies significant structural and metabolic changes in SGCs during PDPN and highlights the critical role of GalCer in maintaining DRG integrity and neuron–glia interactions. By linking GalCer depletion to SGC dysfunction and neuropathic changes, our findings provide a foundation for future research into targeting lipid metabolism as a therapeutic strategy for DPN. Expanding the scope to include other DRG cell types and validating these findings in human-relevant models will further elucidate the complex cellular and molecular mechanisms underlying DPN and pave the way for novel treatment approaches.

## Data Availability

The original data presented in the study are openly available in the National Center for Biotechnology Information (NCBI) Gene Expression Omnibus (GEO) database at GSE176017.

## Supplementary Materials

The following supporting information can be downloaded at: https://www.mdpi.com/article/10.3390/cells14060393/s1, Figure S1: Expression of specific genes in DRG; Table S1: Detailed cell marker; Table S2: Detailed GalCer concentrations in LC-MS/MS.

## Abbreviations

The following abbreviations are used in this manuscript:

ANOVA	Analysis of variance
ATP	Adenosine triphosphate
CCA	Canonical correlation analysis
cDNA	Complementary DNA
DEGs	Differentially expressed genes
DM	Diabetes mellitus
DPN	Diabetic peripheral neuropathy
DRG	Dorsal root ganglion
GalCer	Galactosylceramide
GEO	Gene Expression Omnibus
GFAP	Glial fibrillary acidic protein
GO	Gene Ontology
HBSS	Hanks’ Balanced Salt Solution
HHL	Heat hyperalgesia latency
KEGG	Kyoto Encyclopedia of Genes and Genomes
LC-MS/MS	Liquid chromatography-tandem mass spectrometry
MA	Mechanical allodynia
NCBI	National Center for Biotechnology Information
NS	No significant
PBS	Phosphate-buffered saline
PCA	Principal component analysis
PDPN	Painful diabetic peripheral neuropathy
PSGC	Proliferating satellite glial cells
PWT	Paw withdrawal threshold
qPCR	Quantitative Polymerase Chain Reaction
scRNA-seq	Single-cell RNA sequencing
SGC	Satellite glial cells
SNN	Shared nearest neighbor
SPF	Specific pathogen-free
STZ	Streptozotocin
t-SNE	t-distributed stochastic neighbor embedding
UGT8	UDP-galactose ceramide galactosyltransferase
UMAP	Uniform manifold approximation and projection
UMI	Unique molecular identifier
VECs	Vascular endothelial cells
WL	Withdrawal latency
