# Supplementary material for: Role of Galactosylceramide Metabolism in Satellite Glial Cell Dysfunction and Neuron–Glia Interactions in Painful Diabetic Peripheral Neuropathy
Source: Cells. 2025 Mar 7;14(6):393. doi: 10.3390/cells14060393 (PMC11940725; doi:10.3390/cells14060393)
Supplement: Supplementary file 1 [file cells-14-00393-s001.zip › Supplementary Figrue.pdf]

cells

MDPI

Article

Role of Galactosylceramide Metabolism in Satellite Glial Cell Dysfunction and Neuron-Glia Interactions in Painful Diabetic Peripheral Neuropathy

1. Supplementary Figure

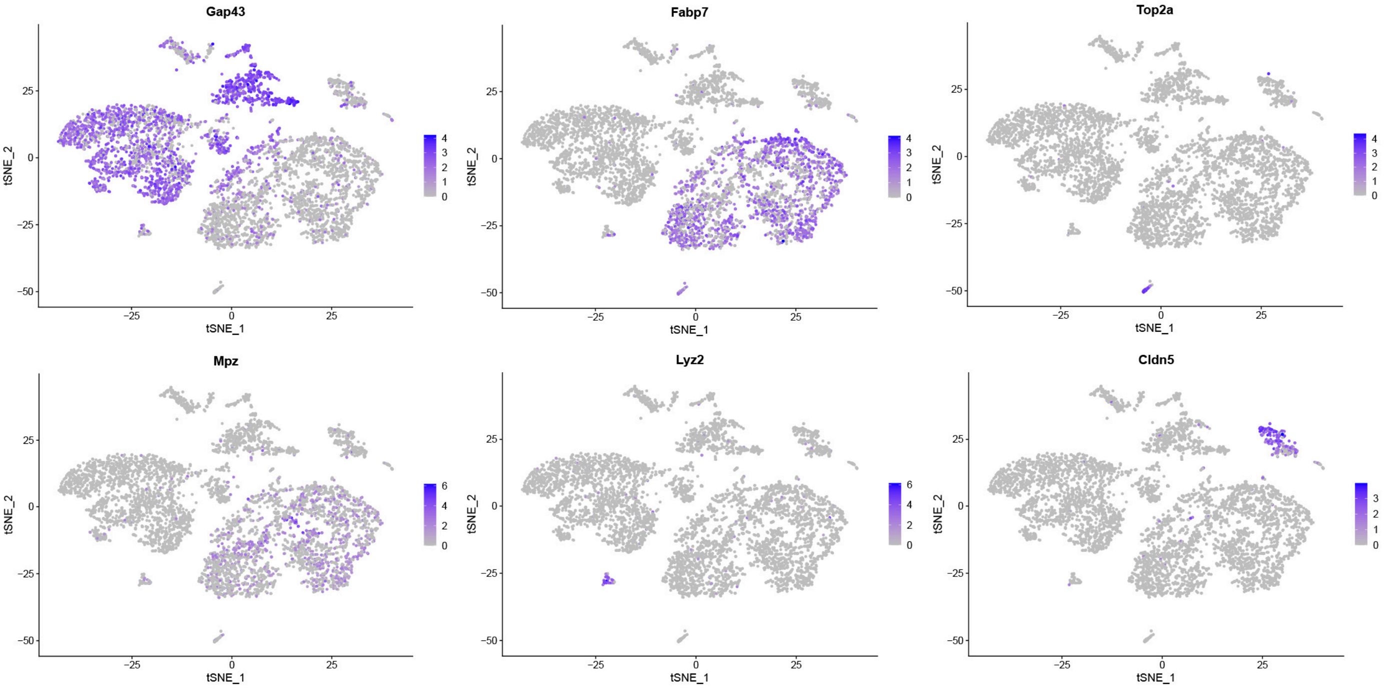

Figure S1. Expression of specific genes in DRG. Feature plots showing the expression of specific genes (*Gap43, Fabp7, Top2a, Mpx, Lyz2, Cldn5*) across the t-SNE plot. The color intensity represents the expression levels of the genes in different cells.
